# The Response of Selected *Triticum* spp. Genotypes with Different Ploidy Levels to Head Blight Caused by *Fusarium culmorum* (W.G.Smith) Sacc.

**DOI:** 10.3390/toxins8040112

**Published:** 2016-04-15

**Authors:** Marian Wiwart, Elżbieta Suchowilska, Wolfang Kandler, Michael Sulyok, Urszula Wachowska, Rudolf Krska

**Affiliations:** 1 Department of Plant Breeding and Seed Production, University of Warmia and Mazury in Olsztyn, pl. Łódzki 3, 10-727 Olsztyn, Poland; ela.suchowilska@uwm.edu.pl; 2 Center for Analytical Chemistry, Department for Agrobiotechnology (IFA-Tulln), University of Natural Resources and Life Sciences, Vienna (BOKU), Konrad Lorenz Str. 20, 3430-Tulln, Austria; wolfgang.kandler@boku.ac.at (W.K.); michael.sulyok@boku.ac.at (M.S.); rudolf.krska@boku.ac.at (R.K.); 3 Department of Entomology, Phytopathology and Molecular Diagnostics, University of Warmia and Mazury in Olsztyn, ul. Prawochenskiego 17, 10-721 Olsztyn, Poland; urszula.wachowska@uwm.edu.pl

**Keywords:** wheat, ploidy level, mycotoxins, principal component analysis, *Fusarium* head blight

## Abstract

Several cultivars and pure lines of *Triticum monococcum*, *T. dicoccon*, *T. polonicum*, *T. spelta* and *T. aestivum* were inoculated with *Fusarium culmorum*, the causal agent of *Fusarium* head blight in wheat. During the three-year study, the infection decreased the values of the analyzed yield components: spike weight (by 5.6% to 15.8%), number of kernels per spike (by 2.8% to 11.8%) and one kernel weight (by 8.4% to 10.7%). *T. spelta* was characterized by the weakest average response to infection. The grain from inoculated spikes contained significantly higher concentrations of deoxynivalenol (DON) and its 3-β-d-glucoside (D3G) than control grain. The D3G/DON ratio ranged from 11.4% to 21.4% in control grain and from 8.1% to 11.6% in inoculated grain. The lowest levels of mycotoxins were found in spelt, and the highest in *T. polonicum* lines and Kamut. PCA revealed that the grain of *T. polonicum* was characterized by an entirely different mycotoxin profile. The weakest response to *F. culmorum* infections was noted in *T. spelta*, and the strongest response in *T. polonicum* breeding lines and Kamut.

## 1. Introduction

Consumers have a growing interest in foods that offer high nutritional value and deliver health benefits. Breeders search for new, high-quality cereal cultivars that meet those requirements. The grain of such cultivars should be characterized by satisfactory processing suitability, high nutrient concentrations and low levels of antinutritional factors [1]. The progress in the cultivation of common wheat was achieved mainly in response to farmers’ demand for high-yielding varieties. Breeding for high yield in cereals is difficult because most of the yield-forming traits are inherited polygenically. Despite those problems, many wheat breeding programs have been successful around the world [2]. The grain of modern high-yielding wheat varieties is much less abundant in protein [3] and micronutrients in comparison with wheat cultivars grown decades ago [4]. Mycotoxins, in particular those produced by pathogens of the genus *Fusarium*, also pose a significant problem for breeders and food producers [5]. Although significant progress has been made in protecting wheat plants against *Fusarium* infections through chemical control [6,7], integrated plant protection methods [8] and the use of antagonistic organisms [9], the cultivation of resistant cultivars does not deliver satisfactory results. Grain infected by *Fusarium* pathogens is characterized by lower processing suitability [10] because starch endosperm is severely damaged by infections [11]. The rheological properties of flour are less desirable [12]. Wheat’s resistance to *Fusarium* head blight (FHB) is determined polygenically, which implies that wholly resistant (immune) varieties cannot be obtained [13]. The sources of resistance to FHB that are most commonly used in breeding, including cultivars Sumai-3 and Frontana [14,15], and the recently discovered genotypes of Asian origin [16], are characterized by several undesirable traits and have poor agronomical characteristics.

The limited number of effective sources of resistance against FHB pathogens in *T. aestivum* has prompted researchers to analyze other species that are closely related to common wheat. Diploid species such as *Triticum monococcum* L., tetraploid taxa of *Triticum dicoccon* Schrank, *Triticum dicoccoides* (Körn. ex Asch. et Graebner) Schweinf, *Triticum polonicum* L. and *Triticum turanicum* Jakubz., and the hexaploid *Triticum spelta* L. are generally characterized by high processing suitability and satisfactory nutritional value, and they constitute valuable initial material for quality [17,18] and resistance breeding [19]. Selected breeding lines of tetraploid wheat species are highly resistant to *Fusarium* pathogens and accumulate relatively low amounts of mycotoxins in grain [19,20,21]. The toxic effects of *Fusarium* pathogens, which can be attributed to their metabolites that belong to different chemical groups (mainly type A and B trichothecenes, fumonisins, zearalenone and its derivatives), have been widely investigated and described in literature [22,23].

The resistance of *Triticum* species other than common wheat to spike infections caused by *Fusarium* pathogens has been explored by relatively few studies [19,24,25,26], and only *Triticum durum* Desf. has been researched quite extensively [27,28,29,30]. A comprehensive analysis of a variety’s response to pathogens should involve evaluations of yield components and quality parameters of grain. The presence of mycotoxins should also be investigated.

The objective of this study was to evaluate the responses of 14 genotypes of five *Triticum* species characterized by different degrees of ploidy to spike infections caused by *Fusarium culmorum* (W.G.Smith) Sacc.

## 2. Results

### 2.1. Weather Conditions

**Figure 1:**
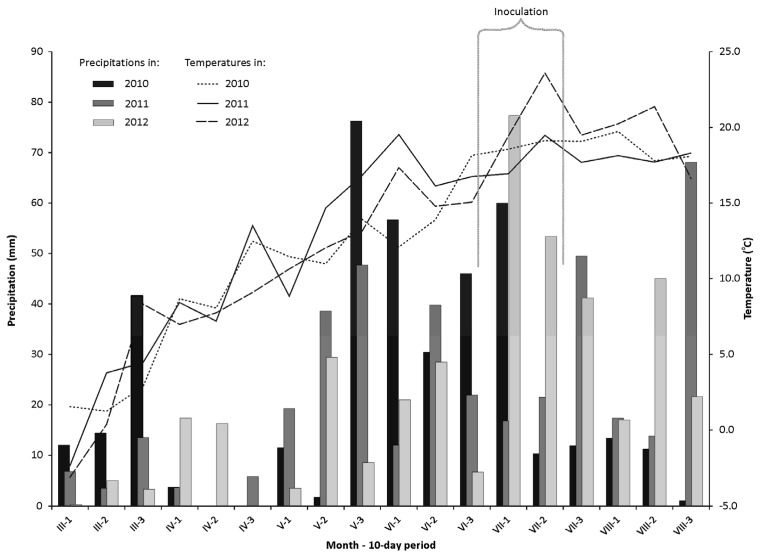
Precipitation and average temperatures in growing seasons of 2010, 2011, and 2012 in the experimental station in Bałcyny.

Field experiments were carried out during three successive years of 2010, 2011 and 2012. Weather conditions (Figure 1) were of critical importance during spike inoculation and in early stages of kernel development (from late June to mid-July). In this respect, significant differences were noted between experimental years. The highest precipitation levels in the first half of July were noted in 2010 and 2012, in late June for 2010, and in mid-July for 2012. In 2012, the highest temperatures were reported between early July and the end of the growing season. Weather conditions were most favorable for spike infections in 2011 and 2012, and least favorable in 2010. Relatively low precipitation levels and high temperatures between late April and early July 2012 created unsupportive growth conditions for the drought-sensitive species *T. aestivum* and *T. monococcum*.

### 2.2. Yield Components

**Figure 2:**
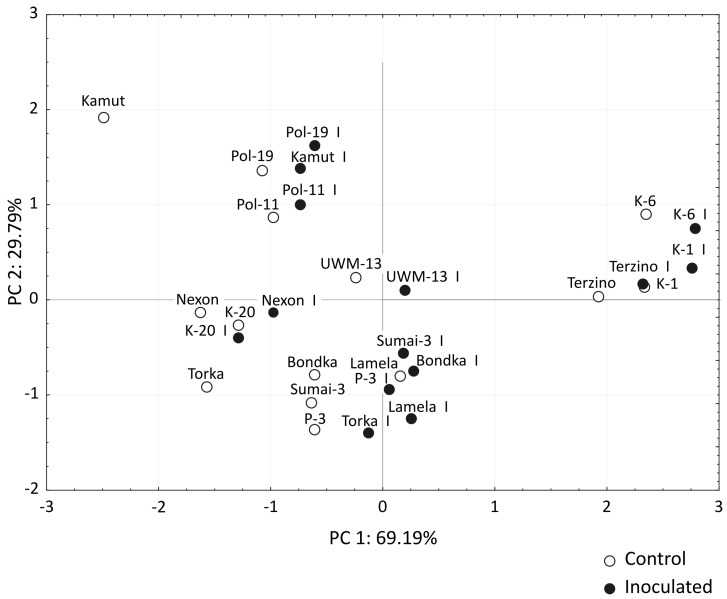
Principal Component Analysis (PCA) results for three biometric parameters of the examined *Triticum* genotypes (mean values for three years). Factor loading values based on the correlations between the Principal Component (PC) (PC1 and PC2, respectively) and spike weight, number of kernels per spike, and one kernel weight are equal: −0.992 and 0.024, −0.665 and−0.743 ,and −0.806 and 0.584, respectively.

The average spike weight, number of kernels per spike and one kernel weight (OKW) of spikes inoculated with *F. culmorum* are presented in Table 1. *F. culmorum* infections decreased the values of the analyzed yield components, mostly OKW and spike weight. Both *T. aestivum* cultivars were characterized by a similar average decrease in spike weight and number of kernels per spike, which did not exceed 20% throughout the experimental period. The noted decrease in OKW values in inoculated spikes of cv. Sumai-3 (2.0%) was more than 12-fold lower than in cv. Torka (25.3%). Both breeding lines of *T. polonicum* and Kamut wheat responded to inoculation with a relatively high drop in the values of the analyzed traits (from 9.5% in OKW to 13.6% in spike weight). The lowest values of the above traits were noted in *T. monococcum* (32.6 mg and 0.9 g, respectively). The F-values produced by the analysis of variance (Table 2) point not only to significant variations (genotype—*G*, inoculation with pathogen—*I*, year of study—*Y*), but also to interactions between experimental factors. All interactions involving the number of kernels per spike and one kernel weight proved to be significant. The above indicates that the applied genotypes not only responded differently to inoculation (*G* × *I*) and were characterized by different values of both traits in successive years (*G* × *Y*), but that those responses differed in successive years (*G* × *I* × *Y*). Interestingly, *G* × *I* and *G* × *Y* interactions were not significant for spike weight. The average values of yield components were analyzed by PCA (Figure 2). The results indicate that the examined genotypes were characterized by significant intraspecific similarities in both control and inoculated treatments. The most distant clusters were observed for *T. monococcum* and *T. polonicum*. Significant similarities were observed between *T. dicoccon* lines and two cultivars of *T. aestivum*.

### 2.3. Mycotoxin Concentrations in Grain

**Figure 3:**
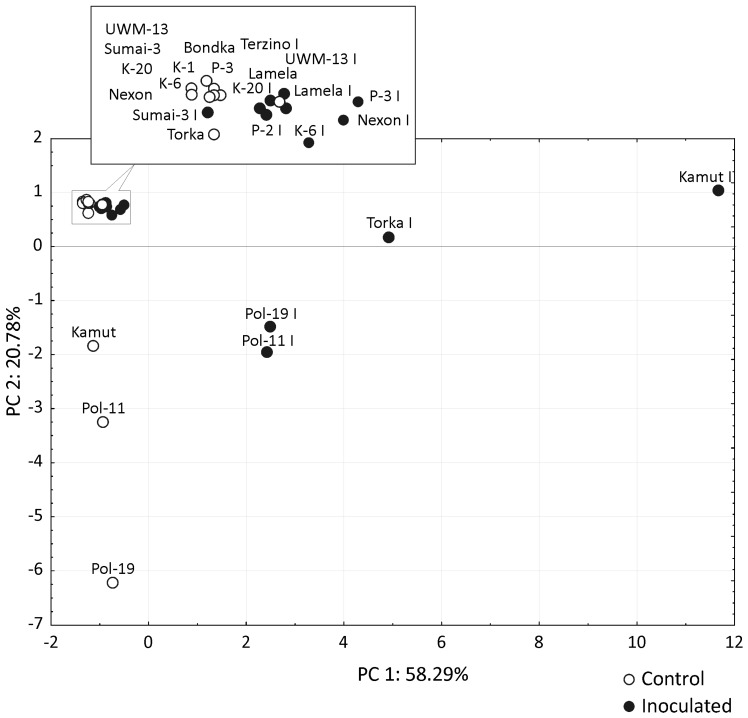
PCA results for mycotoxin concentrations in the grain of *Triticum* genotypes analyzed in 2010. Factor loading values based on the correlation between the Principal Component (PC) and variable (mycotoxin) and contribution of variables are given in Table S1.

**Figure 4:**
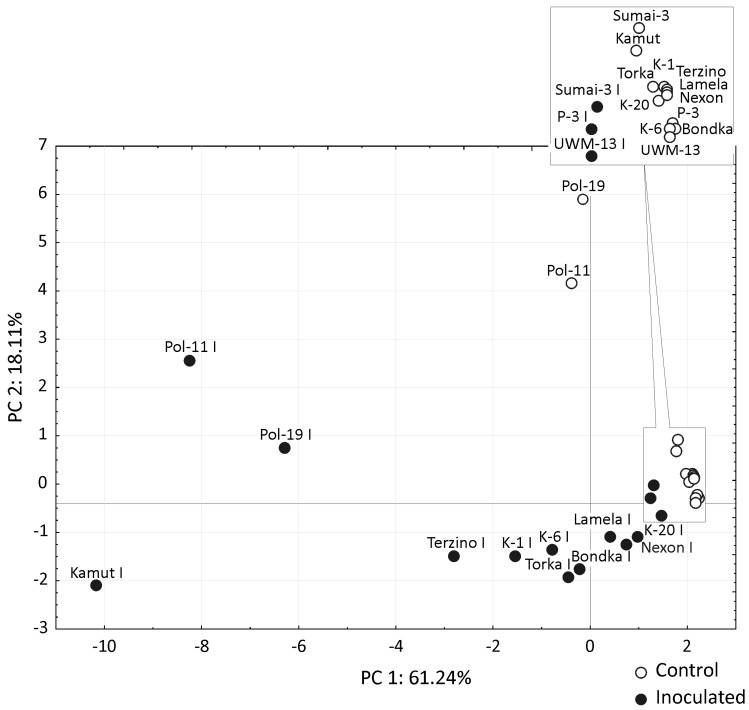
PCA results for mycotoxin concentrations in the grain of *Triticum* genotypes analyzed in 2011. Factor loading values based on the correlation between the Principal Component (PC) and variable (mycotoxin) and contribution of variables are given in Table S1.

The mycotoxin profile was more complex in 2011 (26 metabolites) than in 2010 (21 metabolites), but inoculation with *F. culmorum* led to a dramatic increase in the concentrations of type B trichothecenes, in particular deoxynivalenol (DON), deoxynivalenol-3-β-d-glucopyranoside (D3G) and 3-acetyl-deoxynivalenol (3-Ac DON), in both years (Table 3 and Table 4). In all wheat species except *T. aestivum*, the average DON concentrations in the grain of inoculated spikes were higher in the second than in the first year of the experiment, and more than a three-fold difference was observed in *T. monococcum*. A similar but weaker trend was noted in the concentrations of 3-AcDON. A very strong correlation was observed between DON and D3G levels, and the value of the correlation coefficient *r* reached 0.990 in the first year and 0.953 in the second year of the experiment. In both years, the highest mycotoxin levels were observed in both control and inoculated grain of *T. polonicum*. Interestingly, in addition to very high concentrations of type B trichothecenes and zearalenone (ZEA) produced by *F. culmorum* (52.6 × 10^3^ and 56.1 × 10^3^ μg kg^−1^ in inoculated grain, respectively, and 266 and 1451 μg kg^−1^ in the first and second year of the study, respectively), the grain of *T. polonicum* harvested from control spikes contained considerable amounts of moniliformin (MON) and hexadepsipeptides (3.7 × 10^3^ μg kg^−1^ and 12.9 × 10^3^ μg kg^−1^ in 2010, respectively). The grain of both *T. polonicum* breeding lines and Kamut wheat was also characterized by very high levels of aurofusarin AUF at 41.4 × 10^3^ and 2.0 × 10^3^ μg kg^−1^ in each year of the study, respectively, which were higher than in the grain harvested from the inoculated spikes of the remaining wheat genotypes. The average content of type B trichothecenes in total mycotoxin concentrations was similar in both years of the study in both inoculated grain (75.1% and 77.1%) and control grain (28.9% and 34.7%) (Table 5). The above results suggest that weather conditions significantly influenced mycotoxin concentrations in grain, but had a far less pronounced effect on the relationships between fungal metabolites. Precipitation levels and air temperature differed considerably between 2010 and 2011, in particular in the flowering stage, which affected not only the intensity of *F. culmorum* infections, but also the rate of grain colonization by fungi other than *Fusarium* species. In the group of the examined wheat species, the highest mycotoxin concentrations were noted in the grain of *T. polonicum*. In 2010, fungal metabolite levels in the control grain of Polish wheat were higher than in the grain of the remaining species harvested from inoculated spikes. In 2011, mycotoxin concentrations in *T. polonicum* and the remaining wheat species differed by two orders of magnitude. A comparison of *T. polonicum* and other wheat genotypes indicates that the content of type B trichothecenes in total concentrations of identified toxins was lowest in both control grain (0.6% and 14.8%) and inoculated grain (14.8% and 70%) of *T. polonicum* in both years of the study. A Principal Component Analysis PCA examining the concentrations of all identified mycotoxins revealed completely different fungal metabolite profiles in both *T. polonicum* breeding lines and Kamut wheat and a relatively strong response in cv. Torka, in particular in the first year of the experiment (Figure 3 and Figure 4). In both years of the study, the combined effect of PC1 and PC2 explained more than 79% of variance, which points to high discriminative power. Most importantly, PCA results were similar and comparable in both years.

## 3. Discussion

The responses of 14 diploid, tetraploid and hexaploid *Triticum* genotypes to inoculation with the *F. culmorum* pathogen were presented in this study. The analyzed cultivars (Torka, Nexon, Kamut, Bondka, Lamela, Terzino) are characterized by satisfactory distinctness, uniformity and stability as well as high variety value for cultivation and use, and Sumai-3 is used extensively as a major source of resistance to *Fusarium* head blight FHB.

The responses of wheat species other than *T. aestivum* and *T. durum* to inoculation with pathogens responsible for FHB have been insufficiently studied. The results noted in this study cannot be discussed in depth due to the lack of published data relating to other wheat genotypes. As expected, inoculation contributed to a significant decrease in the values of the three analyzed yield components (in particular OKW), and the variations noted in successive years of the experiment can be attributed to differences in weather conditions that determined the severity of spike infections and the progression of FHB [31]. In 2011, a minor increase in the number of kernels per spike and a simultaneous drop in OKW values were noted in hulled wheat, which can be attributed to low precipitation levels during inoculation and less supportive conditions for the development of infection. In the group of tested cultivars, *T. spelta* was characterized by the weakest average response to inoculation, which is consistent with the results of our previous studies [20]. Spelt’s relatively low susceptibility to infections caused by FHB pathogens could result from long culms and loose spikes which contribute to the lower moisture content of spikelets in comparison with varieties characterized by shorter culms and denser spikes [32]. The above hypothesis was indirectly validated by Mao *et al.* [33] who performed a meta-analysis of quantitative trait locus (QTL) and observed that the presence of *Rht-B1*, *Rht-D1* and *Rht8* dwarfing genes significantly increased susceptibility to FHB, although the above mechanism has not been fully explained.

Wild emmer (*T. dicoccoides*), which is closely related to emmer wheat, is an interesting genetic source that can be potentially used for breeding FHB-resistant cultivars [34]. Due to its genetic similarity to *T. durum*, wild emmer could be a good candidate for breeding research aiming to develop disease-resistant cultivars of durum wheat [35]. Our results indicate that the interactions between experimental factors were significant in many cases. The significance of *G* × *I* interactions could be attributed to differences in the resistance of the analyzed genotypes to the applied pathogen, which indicates that not all tested genotypes are highly suitable for resistance breeding. The significance of *G* × *Y* interactions for the number of grains per spike and one kernel weight is undesirable for breeding because it indicates that both traits are highly influenced by environmental factors. The discussed interaction was not significant for spike weight. The above observations could point to an absence of considerable fluctuations in the yield of the analyzed genotypes under various environmental conditions. However, yield can be influenced by differences in the number of kernels per spike and kernel plumpness.

Although mycotoxin concentrations in grain differed between experimental years, PCA results demonstrated that the analyzed *Triticum* species responded similarly to infection. Very high metabolite levels, in particular AUF and MON, were observed in the grain of *T. polonicum*, which suggests that *T. polonicum* responded completely differently to inoculation with *F. culmorum* or that the analyzed period was characterized by highly supportive conditions for the development of other *Fusarium* pathogens. Aurofusarin, a pigment belonging to the group of dimeric naphthoquinones, is produced by major FHB pathogens, including *F. avenaceum*, *F. culmorum*, *F. graminearum*, *F. sporotrichioides* and *F. tricinctum* [36]. This metabolite is toxic for poultry, in particular turkeys. Moniliformin, produced mainly by *F. avenaceum*, *F. tricinctum*, *F. proliferatum* and *F. subglutinans*, is an inhibitor of several thiamine pyrophosphate-dependent enzymes, glutathione peroxidase and reductase [37]. The lowest content of type B trichothecenes in total mycotoxin concentrations was noted in both control and inoculated grain of *T. polonicum*. The above results could indicate that *T. polonicum* is particularly susceptible to *Fusarium* pathogens that produce MON. *T. polonicum* is a threshable species (with naked grain, similar to common wheat) with long and broad spikelet glumes that can exceed 4 cm. This structural arrangement preserves the high moisture content of spikes and contributes to the development of filamentous fungi, including toxin-producing species. In both years of the study, the maximum level of DON in unprocessed cereals (1,250 μg kg^−1^) [38] was not exceeded in any of the control samples. The presence of D3G, a metabolite of DON, is associated with wheat’s response to FHB pathogens. According to Lemmens *et al.* [39], the ability of wheat lines to convert DON to D3G is linked to quantitative trait locus (QTL) Qfhs.ndsu-3BS that had been previously associated with FHB resistance. The presence of the *Fhb1* gene linked to QTL Qfhs.ndsu-3BS decreases the severity of FHB symptoms and increases the D3G/DON ratio. D3G is characterized by significantly lower toxicity and metabolic activity than DON, but it is partially metabolized by enzymes in the mammalian digestive tract to release DON [40]. A similar process is observed with various intensity during grain fermentation in beer and bread production. The cited authors also noted that the D3G/DON ratio could be a measure of the immune response and the host’s ability to inactivate DON. According to their observations, the D3G/DON ratio can range from 46% to even 70%, although values approximating 20% have been reported in other studies. In our experiment, the average D3G/DON ratio ranged from 11.4% (2011) to 21.4% (2010) in control grain and from 8.1% (2011) to 11.6% (2010) in grain inoculated with *F. culmorum.* Interestingly, the lowest values (up to 9%) were observed in *T. monococcum* that responded strongly to inoculation. The relatively highest values (9.8% to 23%) were reported in *T. spelta*, which was characterized by the weakest average response to *F. culmorum*, and in *T. polonicum* (10.8% to 28.4%) which produced the strongest response to the pathogen. Our findings indicate that the host plant’s ability to produce D3G could be an important, but not the only, defense mechanism for fighting off infections caused by DON-producing pathogens of the genus *Fusarium*.

## 4. Conclusions

The results of this study demonstrate that spelt grain accumulates the lowest amounts of mycotoxins, in particular type B trichothecenes. The analyses of yield-forming traits revealed that spelt was characterized by the weakest response to infection. The strongest responses were noted in *T. polonicum* breeding lines and Kamut wheat, in particular with regard to mycotoxin concentrations in grain, and the above findings were validated by PCA results. PCA supported discrimination between the evaluated genotypes based on their yield-forming traits. Spelt constitutes valuable initial material for breeding new wheat lines characterized by increased resistance to spike infections caused by *Fusarium* pathogens.

## 5. Material and Methods

### 5.1. Material

The experimental material comprised 14 *Triticum* genotypes: two breeding lines and one cultivar of *T. monococcum*, two cultivars and one breeding line of *T. dicoccon*, two breeding lines and one cultivar of *T. spelta*, two breeding lines of *T. polonicum*, and Kamut wheat (Table 6). Two cultivars of *T. aestivum* were also evaluated: Torka, characterized by the highest processing suitability (classified in the group of elite wheat cultivars [41]), and Sumai-3, a widely recognized source of resistance to FHB and a reference cultivar (material obtained from the Nanjing Agricultural University, Nanjing, China). Breeding lines were selected from the extensive collection of the Department of Plant Breeding and Seed Production of the University of Warmia and Mazury in Olsztyn based on at least satisfactory agricultural suitability (yield, grain plumpness, earliness, low susceptibility to lodging). The isolate of *F. culmorum* I_1_ [42] was derived from naturally infected grain of spring wheat grown in northeastern Poland. Pathogenicity and toxin production were checked before the experiment. Species identity and chemotype were additionally confirmed by PCR assays in the Department of Entomology, Phytopathology and Molecular Diagnostics of the University of Warmia and Mazury in Olsztyn.

Genomic DNA was isolated with the use of hexadecyltrimethylammonium bromide (CTAB) [9]. The pathogen was identified to species level under a light microscope (Nikon E 200) based on their sporulation characteristics and the sequence of the ITS 1–5.8SrDNA–ITS 2 region. This fragment was amplified with the use of specific primers ITS5 (F) GTATCGGA CGGAGATCCAGC and ITS4 (R) TTGCTCAGTGCATTG TCGG [43] and the FailSafe PCR system (Epicentre). Chemotypes were differentiated by two primer pairs of TRI13DON F 5′-CATCATGAGACTACTTGTAGTTTGG-3′ and TRI13DONR 5′-GCTAGATCGATTGTTGCATTGAG-3′ as well as TRI13NIVF 5′-CCAAATCCGAAAACCGCA-3′ and TRI13NIVR 5′-TTGAAAGCTCCAATGTCGTG-3′ [44]. The applied isolate belongs to a DON-producing chemotype (synthesizes deoxynivalenol).

### 5.2. Field Experiment

The field experiment was established at the Experimental Station in Bałcyny near Ostróda, Poland (53°36′ N; 19°51′ E) in a slightly undulating area, on Luvisols formed from silty light loam. Local soils are characterized by suitable physicochemical parameters and organic matter content for wheat production. The experiment had a randomized block design with three replications. Plot area was 6 m^2^. Sowing density was 250 germinating kernels per m^2^ for hulled wheats, *T. polonicum* and Kamut, and 360 kernels per m^2^ for common wheat. NPK fertilizer was applied at the pre-sowing rate of 30–25–80 kg ha^−1^. Seeds were not dressed, and Chwastox Extra 300 SL (Organika-Sarzyna S.A., Nowa Sarzyna, Poland, active substance: MCPA, 300 g in 1 L of the product) was used for chemical weed control at 3 L ha^−1^.

Spikes were inoculated by spraying the experimental plots twice with an inoculum suspension of 500,000 conidia per mL in the full flowering stage (BBCH65) [45] at two-day intervals. The suspension was used at a dose of 100 mL per m^2^. Plants from untreated plots served as the control. In the fully ripe stage (BBCH 89), 30 randomly chosen spikes were harvested manually from each inoculated (I) and control (C) plot to measure the average weight of one spike, number of grains per spike and one kernel weight (OKW). Spikelets and grain were harvested with a plot harvester.

### 5.3. Determination of Mycotoxins in Grain

Mycotoxin concentrations in grain were determined according to the method described by Suchowilska *et al.* [20]. Each year, grain samples weighing approx. 1 kg harvested with a plot harvester were collected from each of the field replicates before being pooled into one bulk sample weighing approx. 500 g, which was further subdivided into subsamples. The subsamples of 10 g were ground to fine powder in a laboratory mill (Cullati MFC, Zürich, Switzerland). An amount of 0.5 g of ground sub-samples was extracted with 2 mL of the solvent mixture (acetonitrile/water/acetic acid, 79:20:1, *v*/*v*/*v*). Acetonitrile (LC gradient grade) was supplied by J.T. Baker (Deventer, The Netherlands) and glacial acetic acid (p.a.)—by Sigma-Aldrich (Vienna, Austria). Water was purified successively by reverse osmosis using the Milli-Q plus system from Millipore (Molsheim, France). The samples were extracted for 90 min using the GFL 3017 rotary shaker (GFL, Burgwedel, Germany) and centrifuged for 2 min at 3,000 rpm (15 cm radius) in the GS-6 centrifuge (Beckman Coulter Inc., Fullerton, CA, USA). After centrifugation, the extracts were transferred to glass vials using Pasteur pipettes. Following this, 350 μL aliquots were diluted with the same volume of the dilution mixture (acetonitrile/water/acetic acid, 20:79:1, *v*/*v*/*v*) and directly injected into the LC-MS/MS instrument. For the determination of DON concentrations higher than 500 μg L^−1^ (corresponding to 4 mg kg^−1^ in samples), the raw extract was diluted 1 + 49 (*v* + *v*).

Chromatographic separation was performed at 25 °C in the 1100 Series HPLC System (Agilent, Waldbronn, Germany) equipped with a C_18_ 4 × 3 mm-i.d. security guard cartridge and the Gemini^®^ C_18_ column, 150 × 4.6 mm i.d., 5 μm particle size (Phenomenex, Torrance, CA, USA). Detection and quantification were performed in the Selected Reaction Monitoring (SRM) mode using the QTrap 4000 LC-MS/MS System (Applied Biosystems, Foster City, CA, USA) equipped with a TurboIonSpray electrospray ionization (ESI) source.

For quantification, external calibration was performed using multi-analyte standards prepared and diluted in a 1:1 mixture of extraction and dilution solvent. The results were adjusted for apparent recoveries, which were determined by analyzing a blank wheat sample that had been spiked at one concentration level in triplicate. The relevant values of analytes in the investigated *Triticum* samples were generally in the range of 100% ± 10%, with the exception of D3G (47%), NIV (82%), AOH (69%), AME (69%) β–ZOL (76%).

### 5.4. Statistical Analysis

The results of all measurements and analyses were processed statistically using STATISTICA software (V. 10, StatSoft, Tulsa, OK, USA, 2011) [46]. Normal distribution was checked in the Kolmogorov-Smirnov test, the significance of differences between means was estimated by analysis of variance, and mean values were compared by the Student-Newman-Keuls (SNK) test at *p* < 0.01 or *p* < 0.05. The PCA was performed for two sets of traits: yield components and mycotoxin concentrations.

## Figures and Tables

**Table 1:** The values of three yield components of five *Triticum* sp. taxa inoculated with *F. culmorum* in each year of the experiment (2010–2012).

Species	Treatment	Weight of One Spike (g)			Number of Kernels per Spike			One Kernel Weight (mg)		
		2010	2011	2012	2010	2011	2012	2010	2011	2012
*T. monococcum*	*C*	0.9	0.9	0.9	19.7	20.6	14.3	30.0	29.3	38.6
	*I*	0.9	0.8	0.8	17.2	20.6	10.5	28.4	25.3	35.0
	1 *−* (*I*/*C*) (%)	0	11.1	11.1	12.7	0	26.6	5.3	13.7	9.3
*T. dicoccon*	*C*	1.7	1.9	1.9	32.9	38.4	25.3	31.8	33.6	47.9
	*I*	1.6	1.8	1.5	30.1	39.7	21.1	31.9	30.2	41.1
	1 *−* (*I*/*C*) (%)	5.9	5.3	21.1	8.5	−3.4	16.6	−0.3	10.1	14.2
*T. spelta*	*C*	2.3	1.9	1.8	31.4	33.1	22.1	44.8	40.2	57.2
	*I*	2.1	1.9	1.7	28.5	34.1	22.4	40.5	37.2	55.0
	1 *−* (*I*/*C*) (%)	8.7	0	5.6	9.2	−3.0	−1.4	9.6	7.5	3.8
*T. polonicum*	*C*	1.8	2.3	2.5	21.2	29.3	20.9	50.1	54.6	72.9
	*I*	1.5	2.1	2.3	15.4	27.1	21.6	43.5	48.3	69.1
	1 *−* (*I*/*C*) (%)	16.7	8.7	8.0	27.4	7.5	−3.3	13.2	11.5	5.2
*T. aestivum*	*C*	1.6	2.0	2.3	34.3	41.1	30.6	35.9	38.4	56.5
	*I*	1.3	1.6	1.9	31.6	35.4	28.6	28.1	33.9	49.8
	1 *−* (*I*/*C*) (%)	18.8	20.0	17.4	7.9	13.9	6.5	21.7	11.7	11.9

*C*—control; *I*—inoculation.

**Table 2:** The F-values (with two degrees of freedom) and the relevant probability values (*p*) for the weight of one spike, number of kernels per spike, and one kernel weight in three years of the experiment (2010–2012) under head inoculation with *F. culmorum*.

Source of Variation	Weight of One Spike (g)	Number of Kernels per Spike	One Kernel Weight (mg)
Genotype (*G*)	F _(13, 168)_ = 30.4, *p* < 0.00001	F _(13, 168)_ = 2,085.2, *p* < 0.00001	F _(13, 168)_ = 4,497, *p* < 0.00001
Inoculation (*I*)	F _(1, 168)_ = 3.8, *p* = 0.04925	F _(1, 168)_ = 785.7, *p* < 0.00001	F _(1, 168)_ = 307, *p* < 0.00001
Year (*Y*)	F _(2, 168)_ = 1.1, NS	F _(2, 168)_ = 6,389.4, *p* < 0.00001	F _(2, 168)_ = 1,893, *p* < 0.00001
*G* × *I*	F _(13, 168)_ = 0.29, NS	F _(13, 168)_ = 38.7, *p* < 0.00001	F _(13, 168)_ = 24, *p* < 0.00001
*G* × *Y*	F _(26, 168)_ = 0.79, NS	F _(26, 168)_ = 183.2, *p* < 0.00001	F _(26, 168)_ = 78, *p* < 0.00001
*I* × *Y*	F _(2, 168)_ = 9.2, *p* = 0.0002	F _(2, 168)_ = 164.8, *p* < 0.00001	F _(2, 168)_ = 17,085, *p* < 0.00001
*G* × *I* × *Y*	F _(26, 168)_ = 3.4, *p* < 0.00001	F _(26, 168)_ = 49.0, *p* < 0.00001	F _(26, 168)_ = 292, *p* < 0.00001

NS—not significant.

**Table 3:** Mean toxin concentrations (μg∙kg^−1^) in the grain of five examined *Triticum* species in 2010. Limits of detection (LODs) are given in brackets. If the toxin concentration in a sample was below the LOD, then half of the LOD was used for calculation; ^†^—sum of enniatins (A, A1, A2, B, B1, B2, B3) and beauvericin; #>LOD—number of samples > LOD.

Species	Treatment	Measure	DON (8)	D-3G (1)	3-Ac DON (2)	Deepoxy-DON (15)	NIV (5)	ZEA (2)	ZEA-4 Sulfate (1)	MON (20)	Apicidin (0.04)	Tentoxin (0.2)	AOH (30)	AME (0.8)	AUF (24)	Hexadepsipeptides ^†^
*T. monococcum*	*C*	mean	<LOD	<LOD	<LOD	<LOD	9	<LOD	<LOD	59	<LOD	<LOD	<LOD	<LOD	<LOD	184
		RSD (%)					100			120						100
		#>LOD					1			1						-
	*I*	mean	9.7 × 10^3^	0.9 × 10^3^	334	31	15	7	3	43	<LOD	2	<LOD	<LOD	1.1 × 10^3^	86
		RSD (%)	67	50	74	107	10	81	116	110		132			40	30
		#>LOD	3	3	3	1	3	3	2	2		2			3	-
*T. dicoccon*	*C*	mean	248	32	<LOD	<LOD	6	<LOD	<LOD	14	0.1	0.4	<LOD	<LOD	<LOD	76
		RSD (%)	85	96			101			51	130	133				123
		#>LOD	3	3			1			1	1	1				-
	*I*	mean	10.8 × 10^3^	0.9 × 10^3^	251	<LOD	11	5	3	<LOD	0.4	<LOD	<LOD	<LOD	1.8 × 10^3^	40
		RSD (%)	65	74	52		113	65	80		165				76	25
		#>LOD	3	3	3		2	3	2		1				3	-
*T. spelta*	*C*	mean	135	31	<LOD	<LOD	<LOD	<LOD	<LOD	20	<LOD	1.7	<LOD	<LOD	<LOD	150
		RSD (%)	168	170						88		97				126
		#>LOD	1	1						1		2				-
	*I*	mean	7.0 × 10^3^	1.4 × 10^3^	186	<LOD	12	9	2	27	<LOD	2	<LOD	<LOD	1.7 × 10^3^	83
		RSD (%)	45	24	53		47	143	88	25		99			117	89
		#>LOD	3	3	3		3	2	2	3		1			2	-
*T. polonicum*	*C*	mean	225	64	<LOD	<LOD	55	1	<LOD	3.7 × 10^3^	28.2	0.4	<LOD	<LOD	41.4 × 10^3^	12.9 × 10^3^
		RSD (%)	128	158			86	43		32	91	130			65	41
		#>LOD	3	2			2	1		3	3	1			3	-
	*I*	mean	52.6 × 10^3^	5.7 × 10^3^	594	261	434	266	157	1.5 × 10^3^	3	5	23	27	73.4 × 10^3^	3.4 × 10^3^
		RSD (%)	73	34	93	88	131	39	38	58	84	155	60	171	38	64
		#>LOD	3	3	3	3	3	3	3	3	3	2	1	1	3	-
*T. aestivum*	*C*	mean	<LOD	<LOD	<LOD	<LOD	7	<LOD	<LOD	93	1.3	3.7	<LOD	<LOD	<LOD	71
		RSD (%)					94			126	107	138				101
		#>LOD					1			1	2	1				-
	*I*	mean	35.8 × 10^3^	3.4 × 10^3^	2.2 × 10^3^	165	305	247	32	64	8.0	0.9	<LOD	<LOD	15.1 × 10^3^	121
		RSD (%)	128	106	138	135	131	134	137	54	141	126			135	81
		#>LOD	2	2	2	1	2	2	2	2	1	1			2	-

RSD—relative standard deviation; *C*, *I*—control and inoculated objects; toxin abbreviations—refer to the “Abbreviations” section.

**Table 4:** Mean toxin concentrations (μg∙kg^−1^) in the grain of five examined *Triticum* species in 2011. All explanations and designations as in Table 3.

Species	Treatmnet	Measure	DON	D-3G	3-Ac DON	BUT	NIV	ZEA	ZEA-4 Sulfate	MON	Apicidin	Equisetin	Emodin	Tentoxin	Culmorin	15-Hydroxy-Culmorin	5-Hydroxy-Culmorin	AUF	Chlamydo- sporol	Avenacein Y	Hexadepsi-peptides
			(8)	(1)	(2)	(25)	(5)	(2)	(1)	(20)	(0.04)	(5)	(0.50)	(0.2)	(10)	(10)	(30)	(24)	(1.5)	(15)	-
*T. monococcum*	*C*	Mean	0.26 × 10^3^	10	<LOD	<LOD	<LOD	<LOD	<LOD	67	5.9	21	2	0.6	11	28	<LOD	37	9	<LOD	164
		RSD (%)	36	11						37	79	124	152	73	50	30		22	82		56
		#>LOD	3	3						3	3	2	1	2	2	3		3	3		-
	*I*	mean	30.1 × 10^3^	1.3 × 10^3^	245	2469	33	121	17	122	6.9	33	14	0.7	1,409	2,056	538	3.7 × 10^3^	12	<LOD	245
		RSD (%)	14	11	29	44	11	41	23	55	100	67	139	83	24	31	73	39	163		95
		#>LOD	3	3	3	3	3	3	3	3	3	3	2	2	3	3	3	3	1		-
*T. dicoccum*	*C*	mean	0.52 × 10^3^	30	<LOD	<LOD	<LOD	<LOD	<LOD	16	1.1	<LOD	8	0.7	12	<LOD	<LOD	64	<LOD	<LOD	65
		RSD (%)	94	135						67	64		168	57	100			72			73
		#>LOD	3	3						1	3		1	3	1			2			-
	*I*	mean	20.5 × 10^3^	1.1 × 10^3^	217	769	12	21	3	23	4.2	23	2	0.5	418	932	153	0.8 × 10^3^	<LOD	<LOD	36
		RSD (%)	54	40	40	68	37	79	35	98	17	94	144	76	68	49	51	36			94
		#>LOD	3	3	3	3	-	3	3	1	3	2	1	2	3	3	3	3			-
*T. spelta*	*C*	mean	76	9	<LOD	<LOD	<LOD	<LOD	<LOD	30	5.1	20	3	0.6	<LOD	<LOD	<LOD	71	3	<LOD	100
		RSD (%)	112	90						71	76	134	88	73				38	129		54
		#>LOD	3	3						2	3	2	2	2				3	1		-
	*I*	mean	9.2 × 10^3^	0.9 × 10^3^	141	755	4	26	3	33	2.1	34	2	<LOD	327	609	136	0.9 × 10^3^	3	<LOD	62
		RSD (%)	37	23	45	20	70	47	75	66	80	84	144		23	44	50	53	131		54
		#>LOD	3	3	3	3	1	3	2	2	3	2	1		3	3	3	3	1		-
*T. polonicum*	*C*	mean	1.5 × 10^3^	0.1 × 10^3^	<LOD	233	42	32	1	1507	14.3	275	82	1.6	37	<LOD	<LOD	2.0 × 10^3^	148	1,563	3,570
		RSD (%)	15	44		85	65	137	108	82	108	150	115	93	47			82	76	23	72
		#>LOD	3	3		2	3	2	1	3	3	3	3	2	3			3	2	3	-
	*I*	mean	56.1 × 10^3^	4.8 × 10^3^	342	4,168	121	1,451	103	853	37.3	499	20	1.2	1,875	4,675	909	9.0 × 10^3^	110	479	2,045
		RSD (%)	25	18	15	50	54	28	34	48	55	88	64	36	45	49	45	29	42	42	53
		#>LOD	3	3	3	3	3	3	3	3	3	2	3	3	3	3	3	3	3	3	-
*T. aestivum*	*C*	mean	0.1 × 10^3^	29	<LOD	<LOD	<LOD	<LOD	<LOD	53	1.3	251	2	1.1	11	<LOD	<LOD	56	11	<LOD	176
		RSD (%)	31	10						19	74	138	123	7	79			111	132		73
		#>LOD	2	2						2	2	2	1	2	1			1	1		-
	*I*	mean	10.5 × 10^3^	1.3 × 10^3^	189	888	14	104	5	35	4.2	21	10	0.5	309	994	346	0.7 × 10^3^	7	263	183
		RSD (%)	86	50	98	51	19	66	28	101	27	100	18	110	92	89	122	35	126	137	119
		#>LOD	2	2	2	2	2	2	2	2	2	2	2	2	2	2	2	2	1	1	-

**Table 5:** Mean toxin concentration (μg kg^−1^) in the grain of the examined *Triticum* species in 2010 and 2011.

	Year	Treatment	2010				2011			
Species			Σ Tox	Including		Trich. B/Σ Tox (%)	Σ Tox	Including		Trich. B/Σ Tox (%)
				Trich. B	other			Trich. B	other	
*T. monococcum*		*C*	275	25	250	9.1	659	277	382	42.1
		*I*	14.1 × 10^3^	12.8 × 10^3^	1.2 × 10^3^	91.1	42.4 × 10^3^	31.7 × 10^3^	10.7 × 10^3^	74.7
*T. dicoccon*		*C*	407	300	107	73.7	785	557	228	71.0
		*I*	13.9 × 10^3^	12.0 × 10^3^	1.9 × 10^3^	86.4	25.1 × 10^3^	21.9 × 10^3^	3.2 × 10^3^	87.3
*T. spelta*		*C*	368	182	186	49.5	366	87	279	23.9
		*I*	10.5 × 10^3^	8.7 × 10^3^	1.8 × 10^3^	82.7	13.1 × 10^3^	10.3 × 10^3^	2.9 × 10^3^	78.1
*T. polonicum*		*C*	58.4 × 10^3^	357	58.0 × 10^3^	0.6	11.2 × 10^3^	1,660	9,547	14.8
		*I*	13.8 × 10^4^	59.6 × 10^3^	78.8 × 10^3^	43.1	87.6 × 10^3^	61.4 × 10^3^	26.3 × 10^3^	70.0
*T. aestivum*		*C*	210	25	184	11.9	778	169	609	21.8
		*I*	57.5 × 10^3^	41.8 × 10^3^	15.6 × 10^3^	72.8	15.9 × 10^3^	12.0 × 10^3^	3.9 × 10^3^	75.6
Mean		*C*	11.9 × 10^3^	178	11.7 × 10^3^	28.9	2.8 × 10^3^	550	2,209	34.7
		*I*	57.3 × 10^3^	24.7 × 10^3^	32.6 × 10^3^	75.1	36.8 × 10^3^	27.5 × 10^3^	9.4 × 10^3^	77.1

Σ Tox—sum of all detected mycotoxins; Trich. B—group B trichothecenes.

**Table 6:** Genotypes of *Triticum* sp. examined in the experiment.

Species	Line/Cultivar	Origin
*T. monococcum*	K-1	National Centre for Plant Genetic Resources, Radzików, Poland
	K-6	National Centre for Plant Genetic Resources, Radzików, Poland
	cv. Terzino	Getreidezüchtung Karl Josef Müller, Darzau, Germany
*T. dicoccon*	cv. Lamela	Department of Plant Breeding and Seed Production, University of Warmia and Mazury in Olsztyn, Poland
	cv. Bondka	Department of Plant Breeding and Seed Production, University of Warmia and Mazury in Olsztyn, Poland
	P-3	National Germplasm Resources Laboratory, USA
*T. spelta*	K-20	Leibniz-Institut für Pflanzengenetik und Kulturpflanzenforschung, Gatersleben, Germany
	UWM-13	Breeding strain from the Department of Plant Breeding and Seed Production, University of Warmia and Mazury in Olsztyn, Poland
	cv. Nexon	University of Saskatchewan, Canada
*T. polonicum*	Pol-11	National Germplasm Resources Laboratory, USA
	Pol-19	National Germplasm Resources Laboratory, USA
	cv. Kamut *	Kamut International Ltd., USA
*T. aestivum*	cv. Torka	Plant Breeding Strzelce Ltd., Poland
	cv. Sumai-3	Nanjing Agricultural University, China

* Kamut is classified as *T. turgidum* ssp. *turanicum*, a close relative of *T. turgidum* ssp. *polonicum*. Sowing material supplied by Kamut Int. Ltd., was purchased in a supermarket in Austria.

## Supplementary Material

The following are available online at www.mdpi.com/2072-6651/8/4/112/s1, Table S1: Factor loading values based on the correlation between the PC and variable and contribution of variables (in brackets, in percent) for the mycotoxin concentration in grain of examined wheat genotypes in two years of experiment.

## Abbreviations

The following abbreviations are used in this manuscript:

FHB	*Fusarium* head blight
OKW	one kernel weight
PCA	principal component analysis
d.m.	dry matter
DON	deoxynivalenol
D3G	deoxynivalenol-3-β-d-glucopyranoside
NIV	nivalenol
3-AcDON	3-acetyl-deoxynivalenol
Deepoxy-DON	de-epoxy-deoxynivalenol
ZEA	zearalenone
ZEA-4	sulfate-zearalenone 4-sulfate
MON	moniliformin
AOH	alternariol
AME	alternariol monomethyl ether
AUF	aurofusarin
BUT	butenolide
