# Supplementary material for: The Response of Selected Triticum spp. Genotypes with Different Ploidy Levels to Head Blight Caused by Fusarium culmorum (W.G.Smith) Sacc
Source: Toxins (Basel). 2016 Apr 15;8(4):112. doi: 10.3390/toxins8040112 (PMC4848638; doi:10.3390/toxins8040112)
Supplement: Supplementary file 1 [file toxins-08-00112-s001.pdf]

Supplementary Materials: The Response of Selected *Triticum* spp. Genotypes with Different Ploidy Levels to Head Blight Caused by *Fusarium culmorum* (W.G.Smith) Sacc.

Marian Wiwart [1,*], Elżbieta Suchowilska [1], Wolfang Kandler [2], Michael Sulyok [2], Urszula Wachowska [3] and Rudolf Krska [2]

Table S1. Factor loading values based on the correlation between the Principal Component (PC) and variable (mycotoxin) and contribution of variables (in brackets, in percent) for the mycotoxin concentration in grain of examined wheat genotypes in two years of experiment.

Year / Mycotoxin	2010		2011	
	**PC 1**	**PC 2**	**PC 1**	**PC 2**
DON	0.977 ** (11.7)	0.055 (0.1)	–0.909 ** (7.8)	0.353 (2.7)
D-3G	0.932 ** (10.6)	–0.005 (0.0)	–0.905 ** (7.7)	0.214 (1.0)
3-Ac DON	0.595 * (4.3)	0.061 (0.1)	–0.743 ** (5.2)	0.459 * (4.6)
Deepoxy-DON	0.989 ** (12.0)	0.036 (0.0)	<LOD	
BUT	<LOD		–0.908 ** (7.7)	0.313 (2.1)
NIV	0.964 ** (11.4)	0.020 (0.0)	–0.908 ** (7.7)	–0.004 (0.0)
ZEA	0.803 ** (7.9)	–0.071 (0.2)	–0.922 ** (8.0)	0.039 (0.0)
ZEA-4 Sulfate	0.936 ** (10.7)	–0.072 (0.2)	–0.940 ** (8.3)	0.128 (0.4)
MON	0.061 (0.0)	–0.967 ** (32.2)	–0.455 (1.9)	0.847 ** (15.6)
Apicidin	0.040 (0.0)	–0.881 ** (26.7)	–0.810 ** (6.2)	–0.279 (1.7)
Equisetin	<LOD		–0.519 * (2.5)	–0.450 (3.8)
Emodin	<LOD		–0.085 (0.1)	–0.249 (1.0)
AOH	0.851 ** (8.9)	0.126 (0.5)	<LOD	
AME	0.851 ** (8.9)	0.126 (0.5)	<LOD	
Tentoxin	0.851 ** (8.9)	0.126 (0.5)	–0.325 (1.0)	–0.709 ** (10.9)
Culmorin	<LOD		–0.837 ** (7.0)	0.433 (3.9)
15-Hydroxy-Culmorin	<LOD		–0.884 ** (7.6)	0.408 (3.4)
5-Hydroxy-Culmorin	<LOD		–0.837 **	0.427

			(7.1)	(3.2)
AUF	0.614 *<br>(4.6)	–0.407<br>(5.7)	–0.947 **<br>(8.4)	–0.095<br>(0.2)
Chlamydosporol	<LOD		–0.562 *<br>(3.0)	–0.766 **<br>(12.6)
Avenacein Y	<LOD		–0.296<br>(0.8)	0.882 **<br>(16.5)
Hexadepsipeptides	–0.008<br>(0.0)	–0.982 **<br>(33.2)	–0.455<br>(1.9)	0.866 **<br>(16.3)

*, **—significant at $p < 0.05$ and 0.01, respectively; toxin abbreviations—see "Abbreviations".
